# A Register Study Suggesting Homotypic and Heterotypic Comorbidity Among Individuals With Learning Disabilities

**DOI:** 10.1177/00222194221150230

**Published:** 2023-02-11

**Authors:** Tuija Aro, Reeta Neittaanmäki, Elisa Korhonen, Heli Riihimäki, Minna Torppa

**Affiliations:** 1 University of Jyväskylä, Finland; 2 Niilo Mäki Institute, Jyväskylä, Finland; 3 University of Oulu, Finland; 4 Private practitioner

**Keywords:** learning disabilities, reading disabilities, mathematical disabilities, homotypic and heterotypic comorbidity, register data

## Abstract

The present study examined whether learning disabilities (LD) in reading and/or math (i.e., reading disability [RD], math disability [MD], and RD+MD) co-occur with other diagnoses. The data comprised a clinical sample (*n* = 430) with LD identified in childhood and a sample of matched controls (*n* = 2,140). Their medical diagnoses (according to the *International Classification of Diseases* nosology) until adulthood (20–39 years) were analyzed. The co-occurrence of LD with neurodevelopmental disorders was considered a homotypic comorbidity, and co-occurrence with disorders or diseases from the other diagnostic categories (i.e., mental and behavioral disorders, diseases of the nervous system, injuries, other medical or physical diagnoses) was considered a heterotypic comorbidity. Both homotypic and heterotypic comorbidity were more common in the LD group. Co-occurring neurodevelopmental disorders were the most prominent comorbid disorders, but mental and behavioral disorders, diseases of the nervous system, and injuries were also pronounced in the LD group. Accumulation of diagnoses across the diagnostic categories was more common in the LD group. No differences were found among the RD, MD, and RD+MD subgroups. The findings are relevant from the theoretical perspective, as well as for clinical and educational practice, as they provide understanding regarding individual distress and guiding for the planning of support.

Learning disabilities (LD) are known to be comorbid; that is, individuals with LD are more likely to also have other disorders than their peers without LD. Comorbidity between disorders can be *homotypic* (i.e., between disorders within the same diagnostic grouping) or *heterotypic* (i.e., between disorders from different diagnostic groupings; Angold et al., 1999; see also Moll et al., 2021). Research among individuals with LD has so far focused mainly on the co-occurrence of different subtypes of LD (i.e., reading disability [RD] and math disability [MD]) and on the comorbidity of LD with psychiatric disorders like anxiety or depression (meta-analyses: Nelson & Harwood, 2011a, 2011b; Maag & Reid, 2006) and neuropsychiatric disorders like attention-deficit/hyperactivity disorder (ADHD) or autism spectrum disorders (e.g., DuPaul & Volpe, 2009; Mayes & Calhoun, 2006) among children. In the LD literature, the co-occurrence of RD and MD (RD+MD) is commonly referred to as comorbidity, although in the *Diagnostic and Statistical Manual of Mental Disorders* (*DSM*) and the *International Classification of Diseases* (ICD) both belong to the same diagnostic category of specific learning disorders (*DSM*) or specific developmental disorders of scholastic skills (ICD). Thus, in this study, we do not refer to the co-occurrence of RD and MD as comorbidity but see them and RD+MD as subtypes of LD.

Another active research area has been the co-occurrence of LD and other neurodevelopmental disorders (NDD), such as autism spectrum disorders, ADHD, developmental language disorder, or intellectual disability. This co-occurrence will be considered in the present article as homotypic comorbidity. Much less is known about heterotypic comorbidity (comorbidity of LD and diagnoses from different diagnostic groupings like neurological or physical diseases), although some studies report such findings (see Alabaf et al., 2019; Huscroft-D’Angelo et al., 2019; Mayes & Calhoun, 2006; Shieve et al., 2012). The aim of this study is to add to the knowledge of homotypic and heterotypic comorbidity among individuals with LD.

In the field of LD research, a widely accepted theoretical approach to understanding the underpinnings of co-occurrence of different LD subtypes and other NDDs is the *multiple deficit model* (MDM; McGrath et al., 2020; Pennington, 2006). It posits that multiple predictors probabilistically contribute to NDDs and that shared risk factors contribute to their comorbidity. As a multilevel framework for understanding NDDs, the MDM seeks to cover etiology (genes, environments, and gene–environment interplay), neural processes, neuropsychology, and behavioral symptoms observed among individuals with NDDs. The levels include risk, promotive, and protective factors that are likely to interact across development. Along the same line of thought, stemming from the field of developmental psychology, Rutter (1997) introduced five hypothetical bases for apparent comorbidity. He postulated that comorbidity may represent two manifestations of the same disorder, reflect two stages of the same underlying condition, arise from the same or correlated risk factors, represent a nosologically distinct condition, or be due to one condition predisposing the other. All these bases are plausibly relevant in seeking to understand comorbidity among individuals with LD.

Because the research focus on heterotypic comorbidity among individuals with LD is scarce as of this writing, the delineation of possible causal mechanisms of comorbidity is not yet possible. Thus, descriptive evidence is still needed to feed future LD theory development, as a comprehensive model should consider LD co-occurrence with other disorders or diseases (e.g., Moll et al., 2021). Furthermore, better knowledge about heterotypic comorbidity is of utmost relevance for clinical practice, as it could be of assistance in prevention and intervention planning by helping to understand individual distress and guiding the identification of possible unmet special health care needs and educational requirements. Such knowledge can also provide a perception of the societal and economic consequences of LD.

In the present study, we examined whether there was evidence for homotypic and heterotypic comorbidity in a clinical sample of children with LD followed until adulthood; thus, we aimed to expand the current knowledge by examining the co-occurrence of several types of diagnoses with LD. The diagnoses were categorized into five groups. The category of mental, behavioral, and neurodevelopmental disorders was divided into two subcategories: neurodevelopmental disorders (NDD, excluding LD) and other mental and behavioral disorders. The additional three categories were diseases of the nervous system, injuries, and other medical or physical diagnoses. Comorbidity of LD with other NDDs was considered a homotypic comorbidity due to their supposed neurobiological etiology, whereas the comorbidity with disorders or diseases from the other four categories was considered a heterotypic comorbidity.

Within the diagnostic category of NDDs, the co-occurrence of different LD subtypes (i.e., RD and MD) is well documented (Joyner & Wagner, 2020; Landerl & Moll, 2010; Moll et al., 2014). Several studies also indicate homotypic comorbidity between LD and ADHD (e.g., DuPaul et al., 2013; Willcutt et al., 2007; Willcutt & Pennington, 2000) as well as between LD and developmental language disorder (e.g., Snowling et al., 2020, 2021). Studies analyzing the homotypic comorbidity of LD with other NDDs are fewer, but there is evidence for elevated diagnoses of autism spectrum disorders (ASDs) among individuals with academic problems (Åsberg et al., 2010; Estes et al., 2011; Mayes & Calhoun, 2006). Recently, Hansen et al. (2018) showed high homotypic comorbidity among different NDDs in a sample of patients attending a clinic for child and adolescent psychiatric disorders; approximately 30% of the attendees had one NDD (ADHD, tic disorder, or ASD), and about 20% had more than one NDD. The present study adds to the previous knowledge on homotypic comorbidity by examining the NDD diagnoses until adulthood in a Finnish sample that also allows separation between the subtypes of LD (RD, MD, and RD+MD) and comparison to a large, matched control group.

Heterotypic comorbidity between LD and mental health disorders, such as anxiety (e.g., Nelson & Harwood, 2011b), depression (Nelson & Gregg, 2012), and other psychiatric problems (Cederlöf et al., 2017; Mayes & Calhoun, 2006; Willcutt et al., 2013), is also often reported, especially among children and adolescents. Mental health disorders have also been reported to be common among children and adolescents with NDDs other than LD. Hansen et al. (2018) found that almost 60% of patients attending the clinic for child and adolescent psychiatric disorders and having NDDs also had comorbid psychiatric disorders, but LD was not studied. Most previous studies have been conducted among children or adolescents, and only a few have reported associations between LD and mental health correlations among adults (Davis et al., 2009; Klassen et al., 2013; Wilson et al., 2009). Moreover, it should be noted that most previous studies have reported mean-level differences between individuals with and without LD on self-ratings of psychological well-being; thus, the findings do not necessarily indicate whether participants had higher rates of *clinical* mental disorders (Maag & Reid, 2006). Furthermore, as previous research has mainly focused on RD or LD without further specification, thus neglecting MD and RD+MD subtypes, we lack knowledge of the heterotypic comorbidity of mental health disorders among the different subtypes of LD. However, using the same sample as in this study, Aro et al. (2019) reported that individuals with MD had more reimbursements for antidepressants than the other LD subtypes. In the present study, the focus is on the clinical-level disorders co-occurring with the three subtypes of LD (RD, MD, and RD+MD) and requiring care or treatment other than medication (e.g., sickness/disability allowance, disability pension).

The heterotypic comorbidity between LD and medical or physical diagnoses and neural diseases have rarely been investigated. Most of the studies examining heterotypic comorbidity between NDDs and other diagnoses have focused on ASD (e.g., Kohane et al., 2012) and ADHD (see review by Instanes et al., 2018). ADHD, for instance, has been reported to co-occur with migraines (Fasmer et al., 2011a, 2011b; Kutuk et al., 2018), asthma (Fasmer et al., 2011b), and psoriasis (Hegvik et al., 2018). Studies specifying findings regarding LD are few, but they suggest a high risk of diverse heterotypic comorbidity (Alabaf et al., 2019; Chiang & Cheng, 2014; Shieve et al., 2012). For instance, Alabaf et al. (2019) reported that children with LD (*n* = 294) had a higher risk of epilepsy (odds ratio [OR] 15) and cancer (OR 3.9), but other medical conditions (e.g., asthma, celiac disease, and diarrhea) were also significantly more common among those with LD than among controls. In their sample, Shieve et al. (2012) found that children with LD or other developmental delays (*n* = 1,955) had the highest ORs for diarrhea/colitis (3.2), seizures (4.7), and stuttering (6.0). In sum, the studies concerning heterotypic comorbidity among participants with NDDs have suggested elevated rates in a broad range of medical or physical disorders and neurological diseases, but the studies are mixed regarding on which specific diagnoses they focused. Furthermore, there is a lack of studies specifically targeting LD and its subtypes as well as studies simultaneously examining several diagnostic categories, including medical or physical and mental health disorders.

## The Present Study

We examined whether LD identified in childhood co-occurred with other diagnoses given until the individual reached adulthood. We utilized a Finnish sample with childhood diagnoses of LD (RD, MD, and RD+MD) whose lifelong medical diagnoses given by a physician were drawn from the archives of the Social Insurance Institution of Finland (Kela) when they were adults (ages 20–39 years). The database included diagnostic codes based on ICD 9 (World Health Organization [WHO], 1978) or ICD10 (WHO, 1992) with a specificity level of three digits. For example, a diagnosis for reading disability has a code of F81.0 (s*pecific reading disorder*, see https://www.icd10data.com/ICD10CM/Codes/F01-F99/F80-F89). The diagnoses were the basis of the allowances and benefits provided by Kela. We categorized the registered data diagnoses into five categories: neurodevelopmental disorders, mental and behavioral disorders, diseases of the nervous system, injuries, and other medical or physical diagnoses. We compared the diagnostic frequencies of individuals with LD to the frequencies of their matched controls. Our more specific research questions were as follows:

**Research Question 1:** Did individuals with LD have more diagnoses than controls (a) across all diagnostic categories and (b) in each of the five diagnostic categories?
**Research Question 2:** Did the three LD subgroups differ from each other?
**Research Question 3:** Did diagnoses accumulate within and across diagnostic categories similarly in the LD group and the control group, that is, were multiple diagnoses within and across categories equally common in both groups?

To answer the first and second questions, we first compared the frequencies of the diagnoses in the LD group (*n* = 430) with those of their matched controls (*n* = 2,140) and compared the three LD subgroups with each other. We then counted the ORs for each of the five diagnostic categories after controlling for age, sex, and parental education, comparing the LD and the control groups to ensure that these factors did not confound the group differences in the diagnostic categories. To answer the third question, we compared the accumulation of diagnoses in the study groups, first within each diagnostic category and then across the categories.

## Method

### Procedure and Participants

The study was a follow-up study based on archived data from individuals who had attended the Clinic for Learning Disorders (CLD) in their childhood for a comprehensive neuropsychological assessment to diagnose an LD. The CLD is a public clinic upheld by the Niilo Mäki Institute (NMI) and Jyväskylä Family Counseling Center, Finland. The CLD has served central Finland since 1985, offering assessment and counseling for children (typically 7–13 years of age) with a specific LD. The service is free for the families, and it does not include other services (e.g., health care and social services); thus, it does not place the families in an advantageous position by providing services not related to LD. The children are referred to the CLD mostly by the Family Counseling Center or school psychologists. There are no formal exclusionary criteria at the CLD, but children with primarily behavioral–emotional problems or broad developmental delays are not referred to the CLD. Parents gave informed consent to use the childhood data for research purposes when their child attended the CLD. Ethical approval for the research was given by the University of Jyväskylä Ethical Committee and NMI had given the institutional consent to use the data.

The childhood data, consisting of information concerning demographics, reading and math test scores, and age, were drawn from the CLD’s database. For the purposes of the present study, we identified in this database the participants who were >20 years of age in 2013 and who performed on a reading and/or math test more than 1.5 *SD* below the age- or grade-level mean. A total of 430 individuals met these criteria. Of these, 133 had RD, 113 had MD, and 184 had RD+MD. The Population Register Center (PRC) identified five control individuals for everyone in the CLD data (*N* = 2,140) and provided contact information and parents’ education for all participants. The controls were matched by year of birth, sex, and place of residence at age 7 years (i.e., beginning of compulsory education) so that they would be likely to attend the same schools and health care services. The register data for diagnostic ICD codes for both the former CLD clients and their matched controls were provided by Kela.

### Measures

#### Measures Used to Define LD

Tests used at the CLD to assess reading and math skills varied over the years (1985–2013); therefore, the definitions of LD were based on the test used at the CLD at the time the child attended the clinic. RD, MD, and RD+MD were identified if the child scored 1.5 *SD* below the age- or grade-level mean in the reading and/or math test completed at the CLD. It should be noted that before referral to the CLD, the learning-related difficulties had been observed by the classroom teacher (or parent) and assessed by the special education teacher, and individually planned and/or intensified educational support had been provided. In case problems persisted, the school psychologist or a decision-making team (i.e., administrators, teachers, psychologists, and the parents) were involved (Björn et al., 2016). If these actions turned out to be insufficient, the child was referred to the CLD. This multitiered framework with systematized assessment and instruction, cyclic support, and individually modifiable instruction closely resembles the Response to Intervention model used in the United States (e.g., Fletcher & Vaughn, 2009). In Finland, a formal diagnosis is not needed for special educational support, and thus the children assessed at the CLD were not given an official diagnosis of reading disorder (ICD Diagnosis Code: F81.0) or mathematics disorder (ICD Diagnosis Code: F81.2).

##### Reading Measures

*Reading disability* was defined based on reading fluency since Finnish is an orthographically transparent language, and most children achieve accurate reading skills in the first grade, after which RD is manifested mainly as dysfluent reading (see Seymour et al., 2003). Measures used at the CLD were text or word-list reading tests developed and normed locally (Misku-Text, ÄRPS, and Markkinat) or nationwide (Lukilasse) in Finland. The Misku-Text (NMI, 1985–2004) is a text-reading task normed for 8- to 12-year-old children. The child’s task is to read a short story aloud as fluently and correctly as possible. The ÄRPS (NMI, 1985–2004) is a word and pseudoword reading test; norms are available for Grades 2 through 4. The Markkinat Word List (NMI, 1985–2004) is normed for 8- to 12-year-old children. It consists of 13 words that the child reads aloud as fluently and accurately as possible. The Lukilasse (Häyrinen et al., 1999), a normed test battery for Grades 1 through 6, contains tests for reading, spelling, and mathematical skills. In the Word Reading subtest, the child reads a list of words aloud. Cronbach’s alphas ranged between .94 and .98, depending on the grade (Häyrinen et al., 1999). Psychometric information is not available for the other tests. In Misku, ÄRPS, and Markkinat, the fluency score is obtained based on the time taken by the child to complete the task, and in Lukilasse, by counting the correctly read words within 2 min.

##### Math Measures

Diagnosis of a math disability was based on one of the following tests used at the CLD. The Kaufman Assessment Battery for Children–Arithmetic Subtest (K-ABC; Kaufman & Kaufman, 1983) includes 38 items assessing ability to count and compute, identify numbers, and understand mathematical concepts. The internal consistency values of the K-ABC arithmetic subtest have been found to be at least .84 among school-age children (Matazow et al., 1991). Local norms are available for Grades 2 through 5 (NMI, 1985–2004). In the RMAT (Räsänen, 1992), normed for Grades 3 through 6, the child is requested to perform as many basic arithmetical operations as possible (max. 55) in 10 min. The Cronbach alpha (.86) and test–retest reliability of the RMAT are good (*r* = .82 for 6-month interval and *r* = .76 for 14 months). The Lukilasse Arithmetics subtest (Häyrinen et al., 1999) consists of basic arithmetic operation tasks. According to the manual, Cronbach’s alpha ranged between .55 and .83, depending on the grade (Häyrinen et al., 1999).

#### The Five Diagnostic Categories

In the register data, the diagnoses were recorded based on the ICD-10 (WHO, 1992) since the beginning of 1996, and before that, based on the ICD-9 (WHO, 1978). For the present study, we used the three-digit diagnostic codes provided in the data to create five diagnostic groups, described below. Grouping was done by first constructing dichotomized (yes/no) variables for all diagnoses and then calculating a morbidity score for each category (sum of diagnoses in each category). It should be noted that doctors do not necessarily code all closely related diagnoses (e.g., anxiety and phobia).

##### Neurodevelopmental Disorders

This group contained 14 diagnostic codes; for instance, intellectual development, developmental speech or language disorders, ASDs, and ADHD. It should be noted that LD was not included, as the official diagnosis of LD is not required in Finland for the provision of special educational support; therefore, only those few individuals requiring any of the above-listed allowances granted by Kela were diagnosed.

##### Mental and Behavioral Disorders

This group consisted of 41 different diagnostic codes. For instance, they contained mood disorders, anxiety, dissociative, stress-related, somatoform, and other nonpsychotic mental disorders, eating disorders, and personality disorders.

##### Diseases of the Nervous System

This group contained 23 different diagnostic codes, such as multiple sclerosis, epilepsy and recurrent seizures, myasthenia gravis, and cerebral palsy.

##### Injuries

This group covered 53 different diagnostic codes, such as poisoning and other consequences of external causes.

##### Other Medical or Physical Diagnoses

For this group there were 281 different diagnostic codes. Examples include diseases of the eye, diseases of the ear, diseases of the circulatory, respiratory, gastrointestinal, genitourinary, and musculosceletal systems, endocrine and nutritional difficulties, perinatal and pregnancy-related conditions, congenital malformations, neoplasms, and infections. It should be noted that if a person had several diagnoses from one category, such as gastrointestinal diseases, it was seen in the data as one diagnosis.

#### Basis for Diagnostic Information

The Kela register data used in this study provide lifelong information on the allowances and benefits granted to each individual and the diagnoses that were the basis for granting them. Reimbursements provided for medication were not used in the present study, as the archive included such information only for a limited range of medications. Thus, the diagnoses used in the present study required health care measures other than medication, and the focus of the study was on long-lasting and severe diseases or disorders, which should be borne in mind when reading and interpreting the findings. The allowances and benefits used in the present study are listed below.

##### Sickness Allowance and Disability Pension

A person can apply for a sickness allowance from Kela as compensation for loss of income due to incapacity to work. It is available after the completion of a specified waiting period. A sickness allowance is paid for up to 300 working days on account of the same disease. If the disease or disability becomes persistent and prevents a person from earning a reasonable living, a disability pension can be applied.

##### Special Care Allowance

This allowance is compensation for loss of income available for a person who is unable to do regular work because of the need to participate in the treatment or rehabilitation of a sick child or child with a disability under the age of 16 years. The diagnostic information is coded for the child.

##### Disability Allowance for Persons Under Age 16 Years

This allowance is intended to provide support in the daily lives of a child with a diagnosed disease or a disability if the child needs greater than normal daily care, attention, and rehabilitation due to the disease or disability for longer than at least 6 months.

##### Disability Allowance

This allowance helps support the daily lives, work, or studies of individuals older than 16 years of age who have a disability or chronic disease that (a) has been diagnosed by a doctor and (b) causes either impaired functional capacity for at least a year or a need for assistance or guidance.

##### Rehabilitation Provided by Kela

Kela provides access to diverse types of rehabilitation to help individuals with a disease or impairment live a full life, continue working, or return to work as well as to support economic security during rehabilitation. All the forms of rehabilitation listed below require a diagnosis of a disability or chronic disease that has been diagnosed by a doctor, and these diagnoses were used in the present study. For children, Kela arranges adaptation training courses during which children and close family members can get support in dealing with the changes caused by a disease or impairment. Kela also pays for multidisciplinary individual rehabilitation in institutions (e.g., for sensory impairments, musculoskeletal disorders, rheumatoid arthritis, and general and neurological rehabilitation), intensive medical rehabilitation (e.g., various therapies) if the disease or impairment causes significant problems with daily activities (e.g., hampers full participation in daycare or school activities), and assistive devices if a child’s disease or impairment is a hindrance to their education. For young persons and adults, Kela pays for vocational rehabilitation if the disease or impairment makes it difficult to choose an occupation, pursue an education, or cope with job demands. Kela also provides rehabilitation allowance for young persons (16–19 years of age) while participating in intensive medical rehabilitation aimed at promoting the ability to study or to find work. In addition, rehabilitative psychotherapy, rehabilitation and adaptation training courses, multidisciplinary individual rehabilitation in a rehabilitation institution, assistive devices, intensive medical rehabilitation, and neuropsychological rehabilitation can be paid by Kela.

#### Demographic Variables

In the present study, the following categorical background variables were used: sex (1 = *male*, *n* = 1,798; 2 = *female*, *n* = 772), age group, and mothers’ education. To secure anonymity in the process of merging the clinical data with the register data, we used age categories instead of exact age, but the exact birth year was used by Population Register Center for matching. The sample was classified into four categories based on the individual’s birth year: 1991–1994 (ages 20–23 years in the year 2014; *n* = 887), 1986–1990 (24–28 years, *n* = 737), 1981–1985 (29–33 years; *n* = 690), and 1975–1980 (34–39 years, *n* = 256). Mother’s education was classified into one of three categories: comprehensive school; vocational school or high school; or polytechnic, university, or doctoral education.

## Results

### Differences Between LD and Control Groups in Diagnoses Frequencies

Table 1 reports the frequencies of diagnoses in the five diagnostic categories for the LD and control groups as well as separately for each LD subgroup (RD, MD, and RD+MD). Altogether, 36.4% of the controls and 51.9% of the LD group participants had diagnoses in the register. Note that these percentages do not include LD diagnoses. Crosstab and chi-square tests indicated that having diagnoses was significantly more common among the LD group in all diagnostic categories except for medical or physical disorders. There were no statistically significant differences between the individual LD subgroups (RD, MD, and RD+MD) in the frequency of diagnosis.

**Table 1.** Diagnosis Frequencies, Percentages, and Group Comparisons Between the LD and Control Groups and Among the LD Subgroups.

Diagnosis	LD group vs. control group				LD subgroups			
	Control^a^ *n* (%)	LD^b^ *n* (%)	Ad j*R*	χ^2^(1)	RD^c^ *n* (%)	MD^d^ *n* (%)	RD+MD^e^ *n* (%)	χ^2^(2)
Neurodevelopmental disorder	55 (2.6)	73 (17.0)	12.5	157.03***	19 (14.3)	17 (15.0)	37 (20.1)	2.26
Mental and behavioral disorders	216 (10.1)	60 (14.0)	2.4	5.57*	23 (17.3)	12 (10.6)	25 (13.6)	2.30
Diseases of the nervous system	44 (2.1)	20(4.7)	3.2	9.93**	9 (6.8)	4 (3.5)	7 (3.8)	1.96
Injuries	147 (6.9)	48 (11.2)	3.1	9.41**	14 (10.5)	9 (8.0)	25 (13.6)	2.31
Other medical or physical diagnoses	508 (23.7)	111 (25.8)	0.9	0.84	34 (25.6)	31 (27.4)	46 (25.0)	0.22
No diagnoses (other than LD)	1,360 (63.6)	207 (48.1)	−6.0	35.74***	64 (48.1)	59 (52.2)	84 (45.7)	1.21

*Note.* LD = learning disability; AdjR = Adjusted Standardized Residuals; RD = reading disability; MD = math disability.

a *n* = 2,140. ^b^*n* = 430. ^c^*n* = 133. ^d^*n* = 113. ^e^*n* = 184.

* *p* < .05. **p < .01. ***p < .001.

The difference between the LD and the control groups was especially pronounced in the NDD: 2.6% of the controls had an NDD diagnosis, whereas the corresponding percentage in the LD group was 6.5 times larger (17%). A closer look at the most typical diagnoses within the NDD category revealed that in the LD group 32 individuals had received a diagnosis for developmental language disorder, which was 43.8% of the LD group participants with NDD and 7.4% of the entire LD group. The corresponding frequency among the controls was 14 individuals, which is 25.5% of the controls with an NDD diagnosis and 0.6% of the total control group. Diagnoses of ADHD were also common in the LD group: 27 individuals, which was 37.0% of the LD participants with NDD and 6.2% of the whole LD group. The corresponding frequency among controls was 10 individuals, which is 18.2% of the controls with an NDD diagnosis and 0.4% of the total control group. The other diagnoses were sporadic. For detailed information on diagnoses, see Table S1 in the online supplemental material.

### Odds Ratios for Diagnoses in the LD and Control Groups

Next, we utilized logistic regression analysis to examine the OR between the LD and the control groups for diagnoses in each of the five diagnostic categories. In addition, we individually included, in succession, age group, gender, and parental education as control variables to see whether these factors influenced the ORs in any of the diagnostic categories. The ORs reported in Table 2 come from four different models. In Model 1, the group was the only independent variable (0 = *Control*, 1 = *LD*). In Model 2, both group and age group (1 = *20–23 years*, 2 = *24–28 years*, 3 = *29–33 years*, 4 = *34–39 years*) were included. In Model 3, group, age group, and gender (1 = *male*, 2 = *female*) were included, and in Model 4 parental education (1 = *comprehensive school*, 2 = *vocational school or high school*, 3 = *polytechnic, university, or doctoral education*) was added so that all four independent variables were included. In Models 2 through 4, the variables were included as dummy variables by using contrasts (indicator contrast provided by SPSS), which creates dummy variables for each category to be compared against a specified reference category.

**Table 2.** Odds Ratios for the LD and Control Groups With and Without Age Group, Gender, and Parental Education as Control Variables.

Diagnosis	Control^a^ *n* (%)	LD^b^ *n* (%)	OR^1^ [CI]	OR^2^ [CI]	OR^3^ [CI]	OR^4^ [CI]
Neurodevelopmental disorder	55 (2.6)	73 (17.0)	7.69 [5.33, 11.11]	7.75 [5.36, 11.20]	7.83 [5.40, 11.33]	7.75 [5.34, 11.27]
Mental & behavioral disorders	216 (10.1)	60 (14.0)	1.44 [1.06, 1.96]	1.45 [1.07, 1.98]	1.45 [1.07, 1.98]	1.42 [1.04, 1.94]
Diseases of the nervous system	44 (2.1)	20(4.7)	2.30 [1.34, 3.95]	2.31 [1.35, 3.97]	2.31 [1.35, 3.97]	2.33 [1.35, 4.01]
Injuries	147 (6.9)	48 (11.2)	1.71 [1.21, 2.42]	1.77 [1.24, 2.52]	1.77 [1.24, 2.53]	1.74 [1.22, 2.50]
Other medical or physical diagnoses	508 (23.7)	111 (25.8)	1.12 [0.88, 1.42]	1.12 [0.88, 1.42]	1.12 [0.88, 1.42]	1.10 [0.87, 1.40]

*Note.* The superscript numbers mark the model number with the following independent variables in the models: 1 = *group*, 2 = *group*, *age group*, 3 = *group, age group*, *gender*, 4 = *group, age group, gender, parental education*. CI = confidence interval; LD = learning disability.

a *n* = 2,140. ^b^*n* = 430.

It was clear that adding the control variables did not change the ORs, indicating that differences in age group, sex, or mothers’ education did not contribute to the LD and control group differences regarding the number of diagnoses. The models suggested that the odds of having NDD diagnoses were almost eight times higher in the LD group than in the control group. For the other diagnostic categories, ORs were clearly lower. However, for diseases in the nervous system, the OR was more than twice as high for the LD group than for the control group, suggesting more than twice as high odds of having diseases in the nervous system the LD group than for the control group. The models were found to fit the data well; chi-square omnibus tests of the model coefficients suggested that the models were sufficient (*p* < .05) in their prediction of diagnoses, except for the Model 1 regarding the category of medical or physical disorders. The Hosmer and Lemeshow test also suggested that the models were sufficiently well fitted with the data, except for Models 3 and 4 for medical or physical disorders.

### Accumulation of Diagnoses Within and Across Diagnostic Categories

We next examined two diagnostic accumulation patterns: accumulation within each diagnostic category and accumulation across diagnostic categories. Within-category accumulation analysis was not possible for the injuries category because it only had one diagnosis type. Table 3 reports the diagnosis accumulation patterns within each diagnostic category and across the categories (see Total row) among the control and LD group participants who had at least one diagnosis. When examined within each diagnostic category, no statistically significant group differences were found between the LD and the control groups in one versus more than one diagnosis within any of the four diagnostic categories. However, having multiple diagnoses in general was more common in the LD group than in the control group, χ^2^(1) = 21.92, *p* < .001. Of the LD group participants who had diagnoses, 121 (54.3%) of 223 had multiple diagnoses, while in the control group, the respective percentage was 36.8%.

**Table 3.** Diagnosis Accumulation Patterns Within the Five Diagnostic Categories for Those Control and LD Group Participants Who Have Diagnoses.

Diagnosis	Control group participants^a^			LD group participants^b^			χ^2^(1)
	Have a diagnosis, *n*	1 Diagnosis, *n* (%) Adj*R*	2 or more diagnoses, *n* (%) Adj*R*	Have a diagnosis, *n*	1 Diagnosis, *n* (%) Adj*R*	2 or more diagnoses, *n* (%) Adj*R*	
Neurodevelopmental disorder	55	41 (74.5) 0.6	14 (25.5) –0.6	73	51 (69.9) –0.6	22 (30.1) 0.6	0.340
Mental and behavioral disorders	216	142 (65.7) 1.8	74 (34.3) –1.8	60	32 (53.3) –1.8	28 (46.7) 1.8	3.103
Diseases of the nervous system	44	40 (90.1) 1.7	4 (9.1) –1.7	20	15 (75.0) –1.7	5 (25.0) 1.7	2.880
Injuries	147	147 (6.9)	—	48	48 (11.2)	—	
Other medical or physical diagnoses	508	387 (76.2) –0.5	121 (23.8) –0.5	111	82 (73.9) -0.5	29 (26.1) 0.5	0.264
Total	780	493 (63.2) 4.7	287 (36.8) –4.7	223	102 (45.7) –4.7	121 (54.3) 4.7	21.922***

*Note.* LD = Learning Disabilities; Adj *R* = adjusted standardized residuals.

a *n* = 2,140. ^b^*n* = 430.

* *p* < .05. **p < .01. ***p < .001.

Accumulation across diagnostic groups was further examined by calculating how many of the participants had multiple diagnoses from more than one diagnostic category. The presence of multiple diagnoses from several diagnostic categories was more common in the LD group than in the control group, χ^2^(1) = 18,09, *p* < .001. Of the LD group participants with any diagnoses (*n* = 223), 146 had diagnoses from only one diagnostic category (including 102 participants with one diagnosis from one category and 44 participants with more than one diagnosis from one category), and 77 had diagnoses from multiple diagnostic categories (34.5% of all LD participants with diagnoses). Of the control group participants with any diagnoses (*n* = 780), 618 had diagnoses from only one diagnostic category (including 493 participants with one diagnosis from one category and 125 participants with more than one diagnosis from one category), and 162 had diagnoses from multiple diagnostic categories (20.8% of all control participants with diagnoses).

A closer examination of the various diagnostic category combinations revealed that for the LD group, the most common diagnostic category combinations were (a) NDD and medical or physical disorders (18/223, 8.1%) and (b) mental and behavioral disorders with medical or physical disorder (10/223, 4.5%). Also, various diagnostic category combinations had one to five participants in each combination. For the control group, the most common diagnostic category combinations were (a) mental and behavioral disorders with medical or physical disorders (48/780, 6.2%) and (b) injuries with medical or physical disorders (35/780, 4.5%). Other combinations were rare.

## Discussion

The present study examined whether ICD-based diagnoses from five diagnostic categories (NDD, mental and behavioral disorders, diseases of the nervous system, injuries, and other medical or physical disorders) co-occurred more frequently among individuals identified with LD in childhood in reading and/or math (i.e., RD, MD, or RD+MD) than among the matched controls. The information concerning the lifelong history of diagnoses from the above categories was drawn from the registers of the Social Insurance Institution of Finland when the participants were adults (20–39 years). Four main findings emerged. First, the LD group had more diagnoses overall, and homotypic comorbidity within the NDD group was especially prominent among them. Second, heterotypic comorbidity was also more common in the LD group than in the control group, except for medical or physical disorders. Third, no major differences emerged between the three LD subtypes in any of the analyses. Fourth, the accumulation of diagnoses across diagnostic categories was more common among the LD group than among the control group, whereas the accumulation of several diagnoses within each diagnostic category was similarly common in both groups.

Our findings showing overall more diagnoses among individuals with a childhood history of LD than among the controls both corroborates findings from previous studies conducted mainly among children or adolescents (e.g., Shieve et al., 2012; Wilson et al., 2009) and provides novel information about heterotypic comorbidity beyond the previously often reported comorbidity with psychiatric disorders (e.g., Nelson & Gregg, 2012; Nelson & Harwood, 2011a, 2011b) or behavioral–emotional problems (e.g., Aro et al., 2022), which have also mainly included samples of children (see, however, Klassen et al., 2013; Wilson et al., 2009). Concerning homotypic comorbidity, we found especially high ORs for comorbid NDDs (homotypic comorbidity with LD), indicating that the participants with LD had a more than seven times higher probability of an additional NDD diagnosis than did the controls. The ORs remained almost the same even after controlling for age, sex, and parental education, indicating that these factors did not explain the significant OR difference between the groups in any of the diagnostic categories. Earlier studies have also shown elevated homotypic comorbidity between LD and other NDDs, such as developmental language disorder (e.g., Snowling et al., 2021) and ADHD (e.g., Willcutt & Pennington, 2000), which were also the most typical co-occurring NDDs in our LD sample.

The novel finding was that heterotypic comorbidity was more common among those with LD than among control participants. In our sample, additional diagnoses of mental and behavioral disorders, diseases of the nervous system, and injuries were more common in the LD group than in the control group, although the ORs were lower than for the NDDs. Only medical or physical disorders were not more common among the LD participants than among the control participants. Corresponding to homotypic comorbidity, the heterotypic comorbidity ORs did not change after controlling for sex, age, or parental education. Such findings have not been shown earlier with an equally large-scale study among adults, although some previous studies have suggested the co-occurrence of LD and various diagnoses among children (e.g., Alabaf et al., 2019; Shieve et al., 2012). The co-occurrence of LD and mental health-related problems has also been shown in a few previous studies among adults (e.g., Wilson et al., 2009) and children (e.g., Nelson & Gregg, 2012; Nelson & Harwood, 2011a, 2011b). Although our results do not provide information on the possible mechanisms by which heterotypic comorbidity emerges, they have practical implications. Professionals working with individuals with LD should be aware of the heterotypic comorbidity early on to be able to provide necessary health care services and support for physical and mental well-being in addition to supports dealing with academic difficulties.

The three LD subtypes were not found to differ with respect to the frequency of other diagnoses in the NDD or other diagnostic categories. Previous studies have seldom analyzed the differences between LD subtypes in either homotypic or heterotypic comorbidity. However, there are some earlier studies among children with LD concerning behavioral–emotional symptoms. Willcutt et al. (2013) found that behavioral–emotional problems were more common among children with RD+MD than among those with RD only or MD only. However, Martínez and Semrud-Clikeman (2004) reported contradictory findings: They found no LD subtype differences in emotional adjustment. It is interesting that, in our sample, the co-occurrence of RD and MD did not increase the probability of either homotypic or heterotypic comorbidity in comparison to RD-only or MD-only groups. It is possible that the flexible special education system in Finland identifies individuals with RD+MD early enough to protect this subgroup, at least from severe mental health problems. Because increased heterotypic comorbidity with mental and behavioral disorders was not identified among individuals with RD+MD compared with single LD groups in the present data, our findings suggest that the mechanisms causing heterotypic comorbidity among individuals with RD and those causing them among individuals with MD are not additive and do not interact with each other.

Our analyses indicated that the accumulation of diagnoses within each diagnostic category was equally common among both groups, but accumulation from different diagnostic categories (accumulative heterotypic comorbidity) was more common among individuals with a childhood diagnosis of LD than among their matched controls. That is, in both groups having a diagnosis from one category equally increased the probability of having another diagnosis from the same category, but diagnoses from several diagnostic categories were more common in the LD group. The finding on accumulative heterotypic comorbidity in the LD group is in line with the results of previous studies on NDDs indicating elevated amounts of diagnoses of mental health problems, physical health/somatic problems, and neural system diseases (Alabaf et al., 2019; Chiang & Cheng, 2014; Hansen et al., 2018; Shieve et al., 2012). However, previous studies have not focused on LD but rather more broadly on the NDDs. Also, their focus has most often been on either co-occurrence within the NDD diagnostic category or comorbidity between NDDs and other mental and behavioral disorders. Hence, to our knowledge, this is the first study to indicate that (a) individuals with LD have an elevated risk also for heterotypic comorbid diagnosis and (b) the probability of them having several comorbid diagnoses from several diagnostic categories is heightened. This has several implications for future research, theoretical development, and clinical practice.

### Theoretical Implications

Previous research aiming to understand co-occurrence among individuals with LD has mainly targeted the shared cognitive prerequisites of MDs and RDs (e.g., Koponen et al., 2020; Peterson et al., 2017). Accordingly, theoretical models such as the MDM have focused on homotypic comorbidity within NDDs (e.g., McGrath et al., 2020; Pennington, 2006). The MDM has enhanced multidisciplinary research and inspired studies in the areas of neuropsychology, behavioral genetics, and brain research in the NDD field. However, the present findings on heterotypic comorbidity call for an extension or expansion of the theoretical models to consider also heterotypic comorbidity, which may reflect elements of shared etiology and/or intertwined developmental and environmental processes between diseases and disorders. Although the mechanisms possibly explaining the elevated co-occurrence of LD and other disorders or diseases are beyond the scope of this study, the findings suggest that researchers in the field should consider different mechanisms that might help to understand the multitude of variations in comorbidity.

It is plausible that all the bases of comorbidity hypothesized by Rutter (1997) need to be considered to explain the heterotypic comorbidities found, and different types of explanations may be true for different diagnoses or subgroups of people. It is reasonable to assume that, at one end of the explanatory continuum, some of the comorbidities reflect a common (neuro)biological basis with shared cognitive deficits, thus being a manifestation of the same disorder (e.g., RD and developmental language disorder). On the other end, some comorbidities may be understood as resulting from intertwined cognitive deficits and societal processes in environments or emotional hindrances encountered during the lifetime (e.g., low level of education, low self-efficacy, or poor health literacy associated with LD). In addition, it is possible that two conditions can co-occur without any relationship between them (nosologically distinct categories). The present findings cannot solve the underlying mechanism for the associations but call for an interdisciplinary perspective elaborating a full explanatory theory of LD and comorbid conditions to cover shared risk, protective and promoting factors, and shared developmental mechanisms. To fill this gap in our understanding, a paradigm shift from single to comorbid deficits, including heterotypic comorbidity, and the use of data covering also adult age in LD studies is necessary. Therefore, future research should not neglect the problems co-occurring with LD or exclude participants with co-occurring problems. Furthermore, longitudinal studies with several subsequent assessments and covering the necessary domains are needed to detect causal effects or disclose developmental processes.

### Limitations

When interpreting and generalizing the findings of this study, it should be noted that the register data only included diagnoses warranting allowances and benefits from the Social Insurance Institution of Finland; therefore, disorders or diseases likely to be treated merely with medication were not included in the present percentages. Furthermore, as the data reflect benefits provided by the Finnish social security system, the results are not epidemiological estimates. The fact that the disorders or diseases treated only with medication (i.e., without allowances or benefits) were not included in the analyses has at least two major implications.

First, as our results provide information on relatively severe disorders, they are likely to be conservative. That is, our prevalence estimates of comorbidity may be underestimations, and it can be assumed that various disorders were underrepresented in our sample. An additional reason for possible underestimation of frequencies of diagnoses and their accumulation is that physicians do not necessarily code all closely related diagnoses. Furthermore, it should be noted that another reason for the conservative findings is that children with primarily behavioral–emotional problems or broad developmental delays were not referred to CLD, and this referral bias may have produced artifacts to the detection of comorbidity concerning mental health-related problems and broader developmental disorders.

Second, the data did not allow scrutiny of some interesting specific hypotheses that have recently been expressed concerning LD and especially medical or physical disorders. For instance, it has been proposed that the comorbidity of LD and diseases of the respiratory system may be due to biological mechanisms such as neuronal migration and cilia functions (e.g., Alabaf et al., 2019; Kere, 2014; Niederhofer, 2011). Thus, several questions that require more detailed diagnostic information and measures tapping into genomic and neurobiological aspects remain. More detailed nosological data—and data tracking possible longitudinal changes in the diagnosis over time—would be needed to answer questions regarding whether some disorders represented an early manifestation of another disorder or whether one disorder would be part of another disorder. It should also be remembered that the data were taken from a clinical sample of adults who in their childhood had attended a clinic specializing in LD. It can be surmised that families seeking out this type of service for their child might also be willing to seek out other types of healthcare. This could have resulted in more diagnoses among this group than the population-based control group. Despite these limitations, the strengths of our study using register data are the inclusion of a large control group, reliable clinical childhood diagnosis for the LD group, having no attrition, and covering multiple comorbidities, including medical comorbidities, and tracking them into adulthood. This allowed novel findings, particularly regarding heterotypic comorbidity in large and representative data.

### Conclusion

Our findings imply that both homotypic and heterotypic comorbidities exist and should be carefully considered when planning prevention and intervention, as well as when arranging social and health care services for individuals with LD. This calls for heightened awareness of the need for a comprehensive diagnostic process and multidisciplinary cooperation in any type of consultation or support provision during school age and beyond. We recommend that pedagogical and health care workers consider both mental health and somatic/physical concerns that could be related to the diseases of the neural system to detect different constellations of comorbidity. The educational, psychological, and social problems possibly aggravated by comorbidities associated with LD may also have consequences for society in the form of unemployment and early retirement, which could perhaps be avoided with proper identification and individually tailored support considering all the needs of the individual with LD early enough.

## Supplemental Material

sj-docx-1-ldx-10.1177_00222194221150230 – Supplemental material for A Register Study Suggesting Homotypic and Heterotypic Comorbidity Among Individuals With Learning Disabilities Click here for additional data file. Supplemental material, sj-docx-1-ldx-10.1177_00222194221150230 for A Register Study Suggesting Homotypic and Heterotypic Comorbidity Among Individuals With Learning Disabilities by Tuija Aro, Reeta Neittaanmäki, Elisa Korhonen, Heli Riihimäki and Minna Torppa in Journal of Learning Disabilities
