# Supplementary material for: A Register Study Suggesting Homotypic and Heterotypic Comorbidity Among Individuals With Learning Disabilities
Source: J Learn Disabil. 2023 Feb 11;57(1):30–42. doi: 10.1177/00222194221150230 (PMC10704890; doi:10.1177/00222194221150230)
Supplement: sj-docx-1-ldx-10.1177_00222194221150230 – Supplemental material for A Register Study Suggesting Homotypic and Heterotypic Comorbidity Among Individuals With Learning Disabilities [file sj-docx-1-ldx-10.1177_00222194221150230.docx]

Supplemental Table S1. *Frequencies of Diagnoses by Disorder Typefor Total Sample, LD Group, and Control Group*

| **Diagnosis** | **ICD-10 or older** | **Total** | **Group** | |
| --- | --- | --- | --- | --- |
|  |  |  | **LD** | **Control** |
| ***Frequency of diagnosis of mental and behavioral disorders*** |  | 276 | 60 | 216 |
| Specific nonpsychotic mental disorders following organic brain damage | 310 |  | 0 (0.0%) | 1 (0.5%) |
| Alcohol-related disorders/Mental and behavioural disorders due to use of alcohol | F10 |  | 2 (3.3%) | 2 (0.9%) |
| Opioid related disorders/Mental and behavioral disorders due to use of opioids | F11 |  | 0 (0.0%) | 2 (0.9%) |
| Other psychoactive substance related disorders/Mental and behavioural disorders due to multiple drug use and use of other psychoactive substances | F19 |  | 0 (0.0%) | 4 (1.8%) |
| Schizophrenia | F20 |  | 2 (3.3%) | 9 (4.2%) |
| Delusional disorders/Persistent delusional disorders | F22 |  | 3 (5.0%) | 5 (2.3%) |
| Brief psychotic disorder/Acute and transient psychotic disorders | F23 |  | 1 (1.7%) | 6 (2.8%) |
| Schizoaffective disorders | F25 |  | 3 (5.0%) | 4 (1.8%) |
| Unspecified psychosis not due to a substance or known physiological condition/Unspecified nonorganic psychosis | F29 |  | 3 (5.0%) | 14 (6.5%) |
| Manic episode | F30 |  | 0 (0.0%) | 2 (0.9%) |
| Bipolar disorder | F31 |  | 7 (11.7%) | 14 (6.5%) |
| Depressive episode | F32 |  | 28 (46.7%) | 93 (43.0%) |
| Major depressive disorder, recurrent/Recurrent depressive disorder | F33 |  | 6 (10.0%) | 24 (11.1%) |
| Persistent mood [affective] disorders | F34 |  | 0 (0.0%) | 5 (2.3%) |
| Phobic anxiety disorders | F40 |  | 3 (5.0%) | 12 (5.6%) |
| Other anxiety disorders | F41 |  | 7 (11.7%) | 32 (14.8%) |
| Obsessive-compulsive disorder | F42 |  | 1 (1.7%) | 2 (0.9%) |
| Reaction to severe stress and adjustment disorders | F43 |  | 6 (10.0%) | 33 (15.3%) |
| Eating disorders | F50 |  | 1 (1.7%) | 2 (0.9%) |
| Sleep disorders not due to a substance or known physiological condition | F51 |  | 2 (3.3%) | 12 (5.6%) |
| Psychic factors associated with diseases classified elsewhere | 316 |  | 0 (0.0%) | 0 (0.0%) |
| Unspecified behavioral syndromes associated with physiological disturbances and physical factors | F59 |  | 0 (0.0%) | 0 (0.0%) |
| Specific personality disorders | F60 |  | 3 (5.0%) | 7 (3.2%) |
| Mixed and other personality disorders | F61 |  | 0 (0.0%) | 2 (0.9%) |
| Gender identity disorders | F64 |  | 0 (0.0%) | 1 (0.5%) |
| Conduct disorders | F91 |  | 3 (5.0%) | 2 (0.9%) |
| Mixed disorders of conduct and emotions | F92 |  | 5 (8.3%) | 6 (2.8%) |
| Emotional disorders with onset specific to childhood | F93 |  | 2 (3.3%) | 4 (1.8%) |
| Disorders of social functioning with onset specific to childhood and adolescence | F94 |  | 1 (1.7%) | 1 (0.5%) |
| Other behavioral and emotional disorders with onset usually occurring in childhood and adolescence | F98 |  | 3 (5.0%) | 13 (6.0%) |
| Special symptoms or syndromes, not elsewhere classified | 307 |  | 5 (8.3%) | 14 (6.5%) |
| Adjustment reaction | 309 |  | 0 (0.0%) | 0 (0.0%) |
| Other cerebral degenerations | 331 |  | 1 (1.7%) | 0 (0.0%) |
| Cannabis-related disorders/Mental and behavioral disorders due to use of cannabinoids | F12 |  | 0 (0.0%) | 1 (0.5%) |
| Other stimulant-related disorders/Mental and behavioral disorders due to use of other stimulants, including caffeine | F15 |  | 1 (1.7%) | 0 (0.0%) |
| Schizotypal disorder | F21 |  | 1 (1.7%) | 0 (0.0%) |
| Unspecified mood [affective] disorder | F39 |  | 0 (0.0%) | 2 (0.9%) |
| Dissociative and conversion disorders | F44 |  | 0 (0.0%) | 1 (0.5%) |
| Somatoform disorders | F45 |  | 0 (0.0%) | 1 (0.5%) |
| Mental and behavioral disorders associated with the puerperium, not elsewhere classified | F53 |  | 0 (0.0%) | 0 (0.0%) |
| Mental disorder, not otherwise specified | F99 |  | 1 (1.7%) | 0 (0.0%) |
| ***Frequency of neurodevelopmental disorders*** |  | 128 | 73 | 55 |
| Mild intellectual disabilities | F70 |  | 5 (6.8%) | 4 (7.3%) |
| Moderate intellectual disabilities | F71 |  | 1 (1.4%) | 8 (14.5%) |
| Severe intellectual disabilities | F72 |  | 0 (0.0%) | 4 (7.3%) |
| Unspecified intellectual disabilities | F79 |  | 1 (1.4%) | 9 (16.4%) |
| Other specified mental retardation | 318 |  | 0 (0.0%) | 3 (5.4%) |
| Specific developmental disorders of speech and language | F80 |  | 32 (43.8%) | 14 (25.4%) |
| Specific developmental disorder of motor function | F82 |  | 3 (4.1%) | 1 (1.8%) |
| Mixed specific developmental disorders | F83 |  | 7 (9.6%) | 2 (3.6%) |
| Pervasive developmental disorders | F84 |  | 8 (11.0%) | 9 (16.4%) |
| Other disorders of psychological development | F88 |  | 0 (0.0%) | 0 (0.0%) |
| Unspecified disorder of psychological development | F89 |  | 1 (1.4%) | 0 (0.0%) |
| Specific delays in development | 315 |  | 18 (24.7%) | 12 (21.8%) |
| Attention-deficit/hyperactivity disorders | F90 |  | 27 (37.0%) | 10 (18.2%) |
| Disorders of social functioning with onset specific to childhood and adolescence | F94 |  | 2 (2.7%) | 0 (0.0%) |
| ***Frequency of diseases of the nervous system*** |  | 64 | 20 | 44 |
| Encephalitis, myelitis, and encephalomyelitis | 323 |  | 0 (0.0%) | 1 (2.3%) |
| Sequelae of inflammatory diseases of central nervous system | G09 |  | 0 (0.0%) | 1 (2.3%) |
| Hereditary ataxia | G11 |  | 1 (5.0%) | 0 (0.0%) |
| Multiple sclerosis | G35 |  | 1 (5.0%) | 0 (0.0%) |
| Epilepsy and recurrent seizures | G40 |  | 9 (45.0%) | 23 (52.3%) |
| Migraine | G43 |  | 0 (0.0%) | 2 (4.5%) |
| Sleep disorders | G47 |  | 1 (5.0%) | 1 (2.3%) |
| Nerve root and plexus disorders | G54 |  | 0 (0.0%) | 3 (6.8%) |
| Hereditary and idiopathic neuropathy | G60 |  | 0 (0.0%) | 0 (0.0%) |
| Myasthenia gravis and other myoneural disorders | G70 |  | 1 (5.0%) | 0 (0.0%) |
| Other and unspecified myopathies | G72 |  | 0 (0.0%) | 1 (2.3%) |
| Cerebral palsy | G80 |  | 5 (25.0%) | 5 (11.4%) |

Note. ICD-10 = *International Classification of Diseases, 10th Revision.* World Health Organization.
